# Boys-Specific Text-Comprehension Enhancement With Dual Visual-Auditory Text Presentation Among 12–14 Years-Old Students

**DOI:** 10.3389/fpsyg.2021.574685

**Published:** 2021-04-09

**Authors:** Maria Jose Alvarez-Alonso, Cristina de-la-Peña, Zaira Ortega, Ricardo Scott

**Affiliations:** ^1^Departamento de Psicología Evolutiva y Psicobiología, Universidad Internacional de la Rioja, Logroño, Spain; ^2^Departamento de Psicología Evolutiva y Didáctica, Universidad de Alicante, Alicante, Spain

**Keywords:** language-comprehension, reading, listening, Secondary-school, gender, Spanish, sex-differences, dual-modality

## Abstract

Quality of language comprehension determines performance in all kinds of activities including academics. Processing of words initially develops as auditory, and gradually extends to visual as children learn to read. School failure is highly related to listening and reading comprehension problems. In this study we analyzed sex-differences in comprehension of texts in Spanish (standardized reading test PROLEC-R) in three modalities (visual, auditory, and both simultaneously: dual-modality) presented to 12–14-years old students, native in Spanish. We controlled relevant cognitive variables such as attention (d2), phonological and semantic fluency (FAS) and speed of processing (WISC subtest Coding). Girls’ comprehension was similar in the three modalities of presentation, however boys were importantly benefited by dual-modality as compared to boys exposed only to visual or auditory text presentation. With respect to the relation of text comprehension and school performance, students with low grades in Spanish showed low auditory comprehension. Interestingly, visual and dual modalities preserved comprehension levels in these low skilled students. Our results suggest that the use of visual-text support during auditory language presentation could be beneficial for low school performance students, especially boys, and encourage future research to evaluate the implementation in classes of the rapidly developing technology of simultaneous speech transcription, that could be, in addition, beneficial to non-native students, especially those recently incorporated into school or newly arrived in a country from abroad.

## Introduction

New electronic devices offer easily accessible possibilities for students to simultaneously listen and read texts, and this may enhance reading comprehension in poor skilled students (Wood et al., 2018), or even in students at risk of exclusion for not knowing the official language, or children with auditory problems (Taufan, 2019).

Fluent understanding of written and audible verbal information is essential for school success. Difficulties in reading and listening lay behind low academic performance (Smagorinsky, 2001; Hornickel et al., 2011; Tierney and Kraus, 2013; Cox et al., 2014).

Modality of presentation refers to the sensor route for information processing, such as visual, auditory, or signed words (Penney, 1989; signed modality was not considered here). Determining the most efficient mode for text presentation (audio, visual text or both simultaneously) has been a subject of psychological and educational research (Wolpert, 1971; Green, 1981; Daniel and Woody, 2010); brain activation neuroimaging studies (Green, 1981; Buchweitz et al., 2009) and eye-tracking analysis (Gerbier et al., 2018; Conklin et al., 2020).

Regarding second language learning (L2), research indicates that reading-while-listening is helpful for comprehension, fluency, and vocabulary acquisition (Chang, 2009; Woodall, 2010; Chang and Millett, 2015). Concerning the effects of dual-modality in native languages, Penney (1989) reviewed a collection of memory experiments where sets of words presented in dual-modality produced enhanced memory recall in comparison to words presented in only one modality. Later, Montali and Lewandowski (1996) found that dual-modality benefited less-skilled students at reading social and science passages. In adults, recall after reading text has been reported to be superior to recall after just listening to text (Green, 1981; Dixon et al., 1982; Lund, 1991; Daniel and Woody, 2010). Daniel and Woody (2010) found a better understanding of texts presented for reading-only than listening and reading simultaneously, in young adults. Similarly, Moreno and Mayer (2002) found that adult students who read while listening showed a better comprehension than those who only listened or those whose text was shown with accompanying animations. On the contrary, several research reports have shown worse text comprehension in dual-modality in adults when reading passages of novels (Moyer, 2011; Rogowsky et al., 2016) multimedia narrations (Craig et al., 2002), or technical documents (Kalyuga et al., 2004).

Factors related to the effect of modality presentation are student diversity, age, executive functions performance, type of task, and variability of levels of difficulty (i.e., novels vs. science passages). For instance, possible benefits of a specific modality might be undetected with the presentation of too simple verbal information, not enough to challenge reading skills to a threshold. On the other hand, dual-modality could represent an excessive cognitive load (Kalyuga et al., 2004) and produce distractions when trying to understand very complex texts for which fluency might be interrupted by, for instance, the need to re-reading some parts.

Complex text information processing requires dedicated attention (Bosse and Valdois, 2009; Posner and Rothbart, 2014). Attention skills are highly variable across students regarding their socioeconomic status (Noble et al., 2005) and cognitive factors such as working memory or executive functions (Verhoeven et al., 2011; McVay and Kane, 2012). All these factors contribute to the high variability in reading comprehension among students but one of the most remarkable differences in reading comprehension is student’s sex. Research and tests on reading comprehension consistently show that girls outperform boys in a wide variety of circumstances (Chiu and McBride-Chang, 2006; Logan and Johnston, 2010). We hypothesized that students with difficulties in reading, especially boys as compared to girls, might be specifically benefited by simultaneous audio-text while normally reading. Thus, we aimed at testing text comprehension in boys and girls with three different presentation modalities (audible text, visual text, or dual-modality) using a considerably complex standardized reading text designed for 12–14 years-old (from 7th to 8th grade) Spanish students (Cuetos et al., 2016).

Importantly, there are no studies on the effect of dual-modality presentation in Spanish. This is a relevant matter because opaque and transparent spelling languages might show different effects of dual-modality on comprehension (Tainturier et al., 2011; Kwok et al., 2017).

## Methods

### Ethics Considerations

The study design was approved by the *Universidad Internacional de la Rioja* Ethics Committee amongst written informed consent obtained from each participant’s legal representative. It was managed according to the criteria set by the declaration of Helsinki and local laws.

### Participants

Participants were recruited from a private school in Madrid (Spain). Initially, a total number of 215 participants (94 boys and 121 girls) were selected from 7th to 8th grade (12–14 years-old) (M = 12.89; *SD* = 0.70). Participants included in the study met the following inclusion criteria: being educated in the designated school, not presenting neurological, sensorial, psychopathological or learning disorders, and not having performed the tasks before. However, during data collection, schools were closed due to the worldwide COVID-19 pandemic, thus, not all the students were able to perform all the tests. Therefore, the final sample included: 215 participants (94 boys and 121 girls) for the text comprehension test (PROLEC-R), 177 participants (77 boys and 100 girls) for the verbal fluency (FAS), and the coding test from the WISC Battery, and 150 participants (66 boys and 84 girls) for the attention test (d2).

### Instruments

#### Reading Comprehension Test From the Assessment Battery of Readers Processes, Revised (PROLEC-R) (Cuetos et al., 2016)

The test includes 4 short texts, 2 expositive, and 2 narrative. For this study one of the expositive texts was chosen. The participants should read (or listen) the text in silence; when they are finished, the researcher asks them to put the text away and answer 10 open inferential questions about it. The test can be administered individually or in group format, in the present study the latter format was chosen. The maximum time to perform this test was 15 min. Correct answers are scored with 1 point and wrong answers are scored with 0 points. The outcome measure used in this study was the mean of correct answers.

#### Verbal Fluency Test FAS (Buriel et al., 2004)

This test was used to assess the “Phonological fluency” and the “Semantic fluency” of the participants. For the Phonological fluency subtest, participants were instructed to generate as many words as possible beginning with letters “F,” “A,” and “S” within a 1 min period for each letter. For the Semantic fluency, participants were instructed to generate as many words as possible belonging to the “fruit and vegetable” and “animals” categories within a 1 min period for each category. In both fluency tests proper nouns such as people’s city and country names, and the same word with a different suffix, were excluded. The outcome measures used in this study were the mean of words proposed for each category.

#### Coding Test From the WISC Battery (Wechsler, 2005)

This test is used to assess processing speed. In this study, according to the sample age, only the B form was used. Participants should write certain symbols below the example numbers. To complete the test, 2 min were allowed. The test can be administered individually or in group format. In the present study the latter format was chosen. Correct answers are scored with 1 point and wrong answers are scored with 0 points. The outcome measure used in this study was the mean of correct answers.

#### Attention Test d2 (Brickenkamp, 2007, Adapted to Spanish by Brickenkamp and Seisdedos-Cubero, 2012)

This test was used to assess selective attention. It consists of 14 lines, each containing 47 characters (“p” and “d” with 1–4 dashes arranged either individually or in pairs above and below the character), in total there are 658 items. The subject is required to scan across the line to identify and to mark all “d” with a total of 2 dashes, either above or below the letter. To complete the test 10 min were allowed. The test can be administered individually or in group format, in the present study the latter format was chosen. The outcome measures used in this study were (TR) the total number of items processed, (TA) the total number of correct answers, (O) the number of errors of omission (d’s with two dashes that were not marked), (C) the number of errors of commission (marked d’s with less or more than 2 sashes or p’s), (TOT) total effectiveness of the test [TR- (O + C)] and (CON) concentration index (TA-C).

*Grades in Spanish language* were also collected to have knowledge of the student’s school performance and their general level of reading and comprehension capacities.

### Procedure

Tests were conducted on different days during January and February 2020. The tests for the assessment of attention (d2), phonological and semantic fluency (FAS), and processing speed (WISC) were conducted in the participant’s own classroom. The text comprehension test was performed in the computer lab. In order to fulfill the aim of the study and measure text comprehension by auditory, visual or dual-modality; some adaptations of the test were necessary. The participants assessed for visual modality should read in silence the text shown in a Microsoft PowerPoint file as a presentation with slides running every 20–25 s (visual modality); the participants assessed for auditory modality listened to the text transcribed using an audio recording played through Microsoft Windows 10 default audio software, with a neutral masculine voice (auditory modality), and for the participants assessed for dual-modality, the two formats were set together. The computers used for the test were prepared as follows: one-third of the computers presented the visual modality, another third presented the auditory modality, and the rest of the computers offered the dual-modality presentation. The participants were asked to bring their own earphones due to hygienic reasons. After the text presentation, participants were addressed to a web link where a form was displayed with the text comprehension questions. They were adapted into a Google Form in which anonymization number, sex, age, class, and presentation modality were also requested. Correction of the test was carried out following the test scoring criteria.

### Data Analysis

In a first step, we tested possible group differences in control variables such as attention, phonological and semantic fluency, and speed of processing. Descriptive statistics including mean, standard deviation and standard error were carried out. Secondly, descriptive analysis for language comprehension modality, including mean, standard deviation, standard error, minimum, maximum and confidence interval; were estimated. Regarding the aim of the study of comparing performance in text comprehension given the presentation modality, ANOVA and multiple comparison tests were accomplished. To check if any possible significant differences among the established groups for text comprehension correlated with differences in the grades of Spanish language, Pearson correlations were performed, and additional ANOVA and multiple comparison tests were conducted. Levene test for homocedasticity among Spanish language performance confirmed variances could be assumed to be the same. Subsequently, to test if gender can determine significant differences among the established groups for text comprehension, new ANOVA and multiple comparison tests were conducted. Significance level was 0.05 for all the analyses. Data analyses were conducted using the IBM^®^ SPSS^®^ Statistics 25 for Windows.

## Results

First, we analyzed the general performance of the sample to control the natural differences between the groups of students. Descriptive analysis of test results among all cognitive tasks applied to the sample was within age average (Supplementary Table 1). ANOVA tests and multiple comparison Bonferroni tests showed that groups did not differ significantly in relation to attention measurements (d2), phonological and semantic verbal fluency (FAS), and speed of processing (WISC subtest Coding) (Supplementary Table 2). The following measurements provided a descriptive statistics overview of cognitive performance in boys and girls separately (Supplementary Table 3). Afterward, mean comparison *t*-tests for independent samples were conducted, revealing a sex difference for all cognitive tasks, however, while girls showed better results in phonological fluency (*p* < 0.05 in the 3 components) and speed of processing (*p* < 0.05), boys had a better performance in the d2 test (*p* < 0.05) (Supplementary Table 4).

**FIGURE 1:**
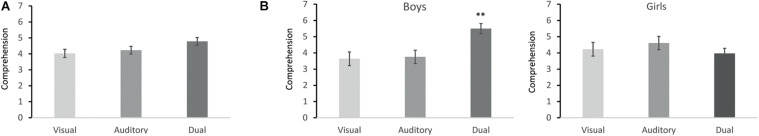
Gender-specific language comprehension for different text presentation modalities. **(A)** Values represent the average score on text comprehension for each experimental group for all students. Error bars correspond to the standard error of the mean (*n* = 73, 74, and 68 for visual, auditory and dual groups). ANOVA analysis showed no significant differences among the groups (*p* = 0.0895). **(B)** Same analysis grouping boys and girls separately (**p < 0.01, Bonferroni among different modalities in boys).

The next step in the analyses was to examine the potential differences in text comprehension depending on the presentation modality (visual, auditory, and dual). Average of comprehension scores showed a non-significant enhancement of comprehension with dual-modality (*F* = 2.44, *p* = n.s.; Table 1 and Figure 1A). When groups were separated by sex, a striking improvement in text comprehension was revealed in boys with dual-modality (Figure 1B; *F* = 8.29, *p* < 0.000). Bonferroni multiple comparison tests showed that text comprehension differed among auditory and dual-modality groups (*p* < 0.005), and between visual and dual-modality groups (*p* < 0.005). On the contrary, not even a small tendency of improvement with dual-modality was found girls (*F* = 0.96, *p* = n.s.; Table 2 and Figure 1B).

**TABLE 1:** Descriptive and mean comparisons of text comprehension in different presentation modality (visual, auditory, and dual).

**Descriptives**								
**Text comprehension**								
	***N***	**Mean**	**Std. deviation**	**Std. error**	**95% Confidence interval for mean**		**Min.**	**Max.**
					**Lower bound**	**Upper bound**		

Visual	73	4.03	2.15	0.25	3.52	4.53	0	8
Auditory	74	4.23	2.08	0.24	3.75	4.71	1	8
Dual	68	4.78	1.99	0.24	4.30	5.26	1	9
Total	215	4.33	2.09	0.14	4.05	4.62	0	9
**ANOVA**								
**Text comprehension**								
	**Sum of squares**		**df**	**Mean square**		***F***	**Sig.**	

Between groups	21.1		2	10.57		2.44	0.089	
Within groups	918.7		212	4.33				
Total	939.8		214					
**Multiple comparisons**								
**Dependent variable: text comprehension**								
**Bonferroni**								
**(I) Presentation modality**	**(J) Presentation modality**	**Mean difference (I-J)**	**Std. error**	**Sig.**	**95% Confidence interval**			
					**Lower bound**		**Upper bound**	

Visual	Auditory	−0.20	0.34	1.000	−1.03		0.63	
	Dual	−0.75	0.35	0.100	−1.60		0.09	
Auditory	Visual	0.20	0.34	1.000	−0.63		1.03	
	Dual	−0.55	0.35	0.352	−1.39		0.29	
Dual	Visual	0.75	0.35	0.100	−0.09		1.60	
	Auditory	0.55	0.35	0.352	−0.29		1.39	

**TABLE 2:** Descriptive and multiple comparisons of text comprehension by presentation modality by sex.

**Boys**								
**Descriptives^a^**								
**Text comprehension**								
	***N***	**Mean**	**Std. deviation**	**Std. error**	**95% Confidence interval for mean**		**Minimum**	**Maximum**
					**Lower bound**	**Upper bound**		

Visual	25	3.64	2.05	0.41	2.79	4.49	0	7
Auditory	33	3.76	2.33	0.40	2.93	4.58	1	8
Dual	36	5.50	1.82	0.30	4.88	6.12	1	9
Total	94	4.39	2.23	0.23	3.94	4.85	0	9
*^*a*^Sex = boys.*								
**ANOVA^a^**								
**Text comprehension**								
	**Sum of squares**		**df**	**Mean square**		***F***	**Sig.**	

Between groups	71.6		2	35.80		8.29	0.000	
Within groups	392.8		91	4.31				
Total	464.4		93					
*^*a*^Sex = boys.*								
**Multiple comparisons^a^**								
**Dependent variable: text comprehension**								
**Bonferroni**								
**(I) Presentation modality**	**(J) Presentation modality**	**Mean difference (I-J)**	**Std. error**	**Sig.**	**95% Confidence interval**			
					**Lower bound**		**Upper bound**	

Visual	Auditory	−0.11	0.55	1.000	−1.46		1.23	
	Dual	−1.86*	0.54	0.003	−3.18		−0.54	
Auditory	Visual	0.11	0.55	1.000	−1.23		1.46	
	Dual	−1.74*	0.50	0.002	−2.96		−0.52	
Dual	Visual	1.86*	0.54	0.003	0.54		3.18	
	Auditory	1.74*	0.50	0.002	0.52		2.96	
*^*a*^Sex = boys.*								
*The mean difference is significant at the 0.05 level.								
**Girls**								
**Descriptives^a^**								
**Text comprehension**								
	***N***	**Mean**	**Std. deviation**	**Std. error**	**95% Confidence interval for mean**		**Minimum**	**Maximum**
					**Lower bound**	**Upper bound**		

Visual	48	4.23	2.19	0.31	3.59	4.87	0	8
Auditory	41	4.61	1.80	0.28	4.04	5.18	2	8
Dual	32	3.97	1.89	0.33	3.29	4.65	1	8
Total	121	4.29	1.98	0.18	3.93	4.65	0	8
*^*a*^Sex = girls.*								
**ANOVA^a^**								
**Text comprehension**								
	**Sum of squares**		**df**	**Mean square**		***F***	**Sig.**	

Between groups	7.67		2	3.83		0.96	0.383	
Within groups	467.20		118	3.95				
Total	474.87		120					
*^*a*^Sex = girls.*								
**Multiple comparisons^a^**								
**Dependent variable: text comprehension**								
**Bonferroni**								
**(I) Presentation modality**	**(J) Presentation modality**	**Mean difference (I-J)**	**Std. error**	**Sig.**	**95% Confidence interval**			
					**Lower bound**		**Upper bound**	

Visual	Auditory	−0.38	0.42	1.000	−1.41		0.65	
	Dual	0.26	0.45	1.000	−0.84		1.36	
Auditory	Visual	0.38	0.42	1.000	−0.65		1.41	
	Dual	0.64	0.46	0.524	−0.50		1.78	
Dual	Visual	−0.26	0.45	1.000	−1.36		0.84	
	Auditory	−0.64	0.46	0.524	−1.78		0.50	
*^*a*^Sex = girls.*								

**FIGURE 2:**
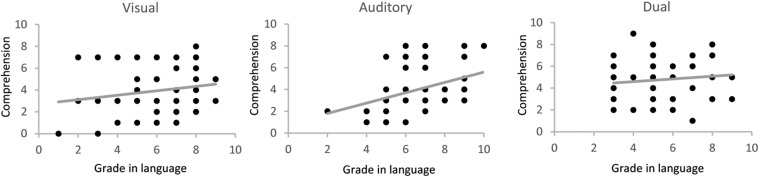
Correlations between grades in language and verbal comprehension for different presentation modalities. Graphs show individual data for text comprehension and grades in Spanish native language. Every dot corresponds to a student with available grades (*n* = 64.53, 59, for visual, auditory, and dual groups). Some dots ovelap. Dotted lines are regression lines fitted to the experimental data with correlation coefficients and *p*-values, respectively, for each group: visual, *r* = 0.163, *p* = 0.19; auditive, *r* = 0.382, *p* = 0.004; dual, *r* = 0.101, *p* = 0.44.

Verbal comprehension in different modalities could be related to student performance at school. Thus, correlations between language comprehension and Spanish language grades (teacher’s scoring) between experimental groups were analyzed. Interestingly, auditory comprehension showed a positive correlation with grades (*r* = 0.38; *p* < 0.005), while visual performance showed just a tendency (*r* = 0.163, *p* < 0.19), and dual comprehension presented a barely flat relation (*r* = 0.101, *p* = n.s.; Figure 2 and Supplementary Figure 1). These results might indicate that low auditory comprehension in low performance students is compensated by visual text support. Remarkably, when descriptives and multiple comparison tests of grades in Spanish among different modalities of text presentation were conducted, the dual-modality group showed significantly lower grades than the auditory group (Bonferroni: *p* = 0.007). However, even in this situation (against our hypothesis because worse lower grades should relate to a decrease, not an enhance, of comprehension) visual support in dual-modality improved comprehension above auditory (which had higher grades) (Supplementary Table 5).

As we found prominent differences between sexes in comprehension with dual-modality (Figure 1), we tested the correlation between text comprehension and grades in language for the three modalities separately in boys and girls. The analysis was suggestive but not conclusive due to the lower number of data with grades available due to the COVID-19 pandemic (see section “Methods”). Boys’ comprehension in auditory modality showed a correlation coefficient of 0.38 with grades, but significance was borderline (*p* = 0.063; Supplementary Figure 1A). Similarly, in girls, the correlation coefficient for auditory modality was 0.30 but, again, not reaching significance (*p* = n.s.; Supplementary Figure 1B). When correlations were performed to examine the relation between sexes and modalities of presentation, they revealed interesting results. While for boys comprehension vs. grades showed a flat correlation (*r* = -0.105; *p* = n.s.), for girls, the correlation coefficient remained similar to auditory modality (*r* = 0.35; *p* = 0.06) (Supplementary Figure 1).

## Discussion

This work aimed to evaluate sex-differences in the comprehension of texts presented in auditive, visual, and dual modalities among 12–14 years-old girls and boys. The main finding is the prominent comprehension enhancement by dual-modality in boys, completely absent in girls. This striking difference between boys and girls might be explained by the faster development of girls (Etchell et al., 2018) and/or by differences in white matter connectivity, such as interhemispheric connectivity (Schmithorst et al., 2008). The finding that girls do not need dual text presentation modality for a normal comprehension could be explained by the observed increase in cognitive scores in girls in verbal fluency and speed of processing, consistent with other studies on this age (Anderson et al., 2001; Dekker et al., 2013) that reveal girls outperforming boys in some cognitive tasks. In addition, speech intelligibility and sentence comprehension in noisy classrooms are superior in 11–12 y-o girls as compared to boys (Prodi et al., 2019).

Intriguingly, our results show that boys perform better in attentional tasks. In dual-modality they must cope with two levels of information at the same time (dual-task), and this might be related to their higher attentional scores reported here. Interestingly, results in bilingual processing indicate that attentional control processing is involved in switching linguistic tasks (Costa et al., 2006), although this tasks-switch was between languages, not between audio/visual versions of the same text.

One of the findings in this work is the loss of positive correlation observed in dual-modality among comprehension and grades in the Spanish language, suggesting that dual-modality might help to compensate poor understanding of texts in students with low grades. This is consistent with several studies on English speakers, reporting that dual-modality aided less-skilled students (Montali and Lewandowski, 1996; Gerbier et al., 2018; Conklin et al., 2020). On the contrary, Rogowsky et al. (2016), did not find differences between dual and single modalities of verbal information processing in adults suggesting that age is relevant for the benefit of dual-modality in language performance, perhaps because it has been further consolidated as compared to children. In addition, the texts used by Rogowsky et al. (2016) were passages of novels, likely less demanding or more interesting than the standardized PROLEC-R used here, designed for the assessment of reading in the specific range of school-age (12–14 y-o).

Skilled readers might be distracted by listening while reading, for instance by forcing a visual or auditive inhibitory control. Our data do not reveal changes in that direction, although a more detailed study focused on good readers would be necessary to rule out the possibility. Our findings suggest that boys could improve speech understanding with the aid of available technology to immediately transcribe spoken text (Arend and Fixmer, 2018; Miner et al., 2020; Nguyen et al., 2020), for instance, on digital screens during teaching sessions. Noticeably, this is what many teachers have been doing traditionally by taking notes on the blackboard while talking (our work would support this classical practice, at least for boys). Obviously, the rapidness of manually transcribing speech on a blackboard is limited and requires additional attention, not always available.

Our results are clear regarding the lack of advantages of dual-modality in girls. However, more research needs to be done to determine whether dual-modality promotes any improvements in girls with low performance in their native language subjects. Nevertheless, even if dual-modality was only helpful for boys, its use in academics should be taken into account, considering the poorer performance of boys as compared to girls at some educational levels (Steinmayr and Spinath, 2008).

Dual-modality benefits are under some debate. In addition to the use of low difficulty texts, previously unnoticed sex-differences, and perhaps age-differences, could explain the controversy. Regarding the age, text comprehension in young adults, men or women, do not seem to be aided by dual-modality, however, interestingly, more complex processing evaluated by transfer tests (which requires the use of text information to solve questions in other contexts) is better with dual-modality in men and worse in women (Flores et al., 2010). This report, together with our results supports the idea that the benefit of dual-modality in boys but not girls depends on age. We have not detected age-related changes in language comprehension, surely because of the short-range of age in our sample. The fact that Flores et al. (2010) detected transfer gender-differences in older subjects suggests that learning and developmental changes compensate for reading difficulties in boys only to some extent.

Friederici (2012) and recently Mossbridge et al. (2017), conducted researches where they predicted the support of cognition in dual or crossmodal visual-auditory signals by enabling the dynamic coordination of inner and sensory processes. This might suggest that receiving information using diverse sensory pathways can enhance performance (Bulkin and Groh, 2006); in our results, the combination of visual displays and auditory information might have improved the performance of the group in general or benefit those students with the worst performance, as the dual-modality may have facilitated the task for them.

The implementation of speech transcription technology in classes would be relatively simple with commercially available software (Google Patents, 2020). However, an effort should be made to adapt a system that allowed (i) quick and easy activation and deactivation when speaking, (ii) integrated display independently of the programs being used during the class, (iii) remote control through a Bluetooth mouse or other device, and (iv) comfortable microphones. Despite these difficulties, the reality is that simultaneous speech transcription is already a reality in many conferences, and it is being further developed for simultaneous translation (Post et al., 2013; Bansal et al., 2017) and even psychological interviews (Miner et al., 2020).

In addition, worldwide changes due to the COVID-19 pandemic have enhanced the exploration of new devices for e-learning platforms and new options for students. Platforms for online teaching frequently lack sound quality, impairing correct understanding of verbal messages at the receptor site. Speech-to-text technology at the transmitter site could greatly contribute to solving this problem.

Moreover, online teaching during the pandemic lockdown in many countries has obliged students to invest a large visual effort at reading the information on screens. In addition to reducing eye strain (Rosenfield and Mcoptom, 2016), our results suggest that at least boys’ reading comprehension would improve by simultaneous audio reading (quickly developing by different companies; i.e., Natural Reader, Nuance, Google, etc.).

Future plans involve adapting already available technology for simultaneous transcription of verbal information during classes and implement this technology at different educational levels from primary to university school, and finally, evaluate academic results, and student/teacher/family perception of these strategies. Additionally, this technology might be advantageous for students non-native in Spanish, especially those recently incorporated to school or newly arrived from abroad. These students might learn the new language faster, integrate more easily in the group and avoid the risk of being academically frustrated and delayed. Although dual-modality facilitation for second language learning has been extensively reported (Brown et al., 2008; Chang and Millett, 2014, 2015), the benefits for inclusion should be tested in natural conditions.

## Limitations

The study was carried out with participants from a single center. Therefore, there may be variables contaminating the results and adversely affecting their generalization. The participants belonged to a middle-high socioeconomic status so the observed better reading performance in girls might not be present in lower levels. Further studies are required to verify this possibility.

Although we have measured the speed of processing with the WISC test, related to intelligence, we cannot rule out that some unexpected differences in intelligence among participants might explain the results to some extent.

Our results show slightly higher attention in some sections of the d2 test which might be related to the different performance of boys and girls in dual-modality. However, such a conclusion would require testing attention in the different modalities.

Attentional performance has been related to switching linguistic tasks (Costa et al., 2006). Another interesting future research would be to investigate the link between dual-modality and switching linguistic tasks.

Regarding the possibility that skilled readers might be forcing a visual or auditive inhibitory control in dual-modality, and therefore being harmed in their comprehension, would require a more detailed study focused on good readers.

A possible limitation of our work is that we used male voice for the auditive and dual modalities. Sex-differences could be related to this, however, voice acoustics differences have been reported to be quite similar among individuals and the general population (Lee et al., 2019). In addition, although differences in brain activity in response to female/male voices have been reported (Lattner et al., 2005), no evidence of differences among genders in auditive language perception with male or female voices have been reported (Mullennix et al., 1995; Lattner et al., 2005). In this work, the auditive text was presented with a male voice only, but indifferently to boys and girls.

## Data Availability Statement

The raw data supporting the conclusions of this article will be made available by the authors, without undue reservation.

## Ethics Statement

The study design was approved by the Universidad Internacional de la Rioja Ethics Committee amongst written informed consent obtained from each participant’s legal representative. It was managed according to the criteria set by the declaration of Helsinki and local laws.

## Author Contributions

Cd-l-P collected the data. MA-A and RS adapted reading comprehension test methodology and wrote the manuscript. Cd-l-P, ZO, and MA-A corrected the filled in tests. MA-A, ZO, and RS analyzed the data. MA-A, Cd-l-P, ZO, and RS designed research. All authors contributed with valuable comments along the research, including analysis and manuscript writing.

## Conflict of Interest

The authors declare that the research was conducted in the absence of any commercial or financial relationships that could be construed as a potential conflict of interest.
