# Supplementary material for: Boys-Specific Text-Comprehension Enhancement With Dual Visual-Auditory Text Presentation Among 12–14 Years-Old Students
Source: Front Psychol. 2021 Apr 9;12:574685. doi: 10.3389/fpsyg.2021.574685 (PMC8062718; doi:10.3389/fpsyg.2021.574685)
Supplement: Supplementary file 1 [file Data_Sheet_1.docx]

**SUPPLEMENTAL MATERIAL (TABLES AND FIGURES)**

**SUPPLEMENTAL TABLES**

**Supplemental Table 1**.

*Descriptives for attention, phonological and semantic fluency and speed of processing in different modalities of text presentation (visual, auditory and dual).*

|  |  |  |  |  |  |  |
| --- | --- | --- | --- | --- | --- | --- |
|  |  |  | N | Mean | Std. Desviation | Std. Error |
| Attention | TOT | visual | 58 | 324.22 | 75.01 | 9.85 |
|  |  | auditory | 48 | 317.04 | 75.29 | 10.86 |
|  |  | dual | 44 | 328.68 | 63.57 | 9.58 |
|  |  | total | 150 | 323.23 | 71.61 | 5.84 |
|  | CON | visual | 58 | 131.15 | 36.06 | 4.73 |
|  |  | auditory | 48 | 123.37 | 47.22 | 6.81 |
|  |  | dual | 44 | 126.02 | 37.05 | 5.58 |
|  |  | total | 150 | 127.16 | 40.10 | 3.27 |
| Phonological and semantic fluency | F | visual | 64 | 9.57 | 3.39 | 0.42 |
|  |  | auditory | 54 | 9.53 | 3.22 | 0.43 |
|  |  | dual | 59 | 8.42 | 3.14 | 0.40 |
|  |  | total | 177 | 9.18 | 3.28 | 0.24 |
|  | A | visual | 64 | 9.18 | 3.24 | 0.40 |
|  |  | auditory | 54 | 9.09 | 2.87 | 0.39 |
|  |  | dual | 59 | 8.20 | 3.08 | 0.40 |
|  |  | total | 177 | 8.85 | 3.09 | 0.23 |
|  | S | visual | 64 | 11.67 | 3.05 | 0.38 |
|  |  | auditory | 54 | 11.43 | 3.57 | 0.48 |
|  |  | dual | 59 | 10.57 | 2.96 | 0.38 |
|  |  | total | 177 | 11.24 | 3.21 | 0.24 |
|  | Fruits | visual | 64 | 11.21 | 2.05 | 0.25 |
|  |  | auditory | 54 | 11.74 | 3.02 | 0.41 |
|  |  | dual | 59 | 11.81 | 2.65 | 0.34 |
|  |  | total | 177 | 11.57 | 2.58 | 0.19 |
|  | Animals | visual | 64 | 16.29 | 2.62 | 0.32 |
|  |  | auditory | 54 | 15.16 | 4.35 | 0.59 |
|  |  | dual | 59 | 15 | 3.69 | 0.48 |
|  |  | total | 177 | 15.51 | 3.61 | 0.27 |
| Speed of processing | Keys | visual | 64 | 57.56 | 12.17 | 1.52 |
|  |  | auditory | 54 | 57.07 | 11.52 | 1.56 |
|  |  | dual | 59 | 58.37 | 12.30 | 1.60 |
|  |  | total | 177 | 57.68 | 11.96 | 0.89 |

**Abbreviations:** TOT. total test effectiveness; CON. concentration index

**Supplemental Table 2.**

*Multiple mean comparisons of attention. phonological and semantic fluency and speed of processing between different groups of presentation modality.*

*ANOVA*

|  |  |  | Sum of Squares | df | Mean Square | F | Sig. |
| --- | --- | --- | --- | --- | --- | --- | --- |
| Attention (d2) | TOT | Between Groups | 3203.2 | 2 | 1601.64 | 0.30 | 0.734 |
|  |  | Within Groups | 760999.5 | 147 | 5176.86 |  |  |
|  |  | Total | 764202.8 | 149 |  |  |  |
|  | CON | Between Groups | 1670.3 | 2 | 835.16 | 0.51 | 0.598 |
|  |  | Within Groups | 238015.8 | 147 | 1619.15 |  |  |
|  |  | Total | 239686.2 | 149 |  |  |  |
| Phonological* and semantic^ fluency | F* | Between Groups | 50.7 | 2 | 25.38 | 2.38 | 0.095 |
|  |  | Within Groups | 1853.4 | 174 | 10.65 |  |  |
|  |  | Total | 1904.2 | 176 |  |  |  |
|  | A* | Between Groups | 29.0 | 2 | 14.54 | 1.53 | 0.219 |
|  |  | Within Groups | 1652.3 | 174 | 9.49 |  |  |
|  |  | Total | 1681.4 | 176 |  |  |  |
|  | S* | Between Groups | 40.6 | 2 | 20.30 | 1.98 | 0.14 |
|  |  | Within Groups | 1775.9 | 174 | 10.20 |  |  |
|  |  | Total | 1816.5 | 176 |  |  |  |
|  | Fruits^ | Between Groups | 12.9 | 2 | 6.48 | 0 .97 | 0.38 |
|  |  | Within Groups | 1160.2 | 174 | 6.66 |  |  |
|  |  | Total | 1173.2 | 176 |  |  |  |
|  | Animal^ | Between Groups | 61.3 | 2 | 30.66 | 2.38 | 0.095 |
|  |  | Within Groups | 2232.8 | 174 | 12.83 |  |  |
|  |  | Total | 2294.1 | 176 |  |  |  |
| Speed of processing | Keys | Between Groups | 49.0 | 2 | 24.51 | 0.17 | 0.844 |
|  |  | Within Groups | 25157.2 | 174 | 144.58 |  |  |
|  |  | Total | 25206.2 | 176 |  |  |  |

**Abbreviations:** TOT. total test effectiveness; CON. concentration index

**Supplemental Table 3.**

*Descriptives for attention, phonological and semantic fluency and speed of processing according to sex.*

|  | | | | | |
| --- | --- | --- | --- | --- | --- |
|  | Sex | N | Mean | Std. Deviation | Std. Error Mean |
| Age | boys | 66 | 12.92 | 0.75 | 0.09 |
|  | girls | 84 | 12.87 | 0.67 | 0.07 |
| Spanish grade | boys | 76 | 5.57 | 1.69 | 0.19 |
|  | girls | 100 | 6.43 | 1.69 | 0.17 |
| Text comprehension | boys | 94 | 4.39 | 2.23 | 0.23 |
|  | girls | 121 | 4.29 | 1.98 | 0.18 |
| F* | boys | 77 | 8.13 | 2.99 | 0.34 |
|  | girls | 100 | 9.99 | 3.28 | 0.32 |
| A* | boys | 77 | 8.32 | 3.14 | 0.35 |
|  | girls | 100 | 9.27 | 2.99 | 0.29 |
| S* | boys | 77 | 10.55 | 3.00 | 0.34 |
|  | girls | 100 | 11.78 | 3.27 | 0.32 |
| Animals* | boys | 77 | 15.04 | 3.45 | 0.39 |
|  | girls | 100 | 15.89 | 3.70 | 0.37 |
| Fruits* | boys | 77 | 11.27 | 2.62 | 0.29 |
|  | girls | 100 | 11.81 | 2.53 | 0.25 |
| Speed Processing | boys | 77 | 55.43 | 12.82 | 1.46 |
|  | girls | 100 | 59.42 | 11.02 | 1.10 |
|  |  |  |  |  |  |
| TR (d2) | boys | 77 | 359.57 | 76.97 | 8.77 |
|  | girls | 100 | 331.63 | 81.65 | 8.16 |
| TA (d2) | boys | 77 | 138.83 | 37.64 | 4.29 |
|  | girls | 100 | 125.20 | 35.08 | 3.50 |
| O (d2) | boys | 77 | 17.65 | 25.32 | 2.88 |
|  | girls | 100 | 17.90 | 28.66 | 2.86 |
| C (d2) | boys | 77 | 6.90 | 20.58 | 2.34 |
|  | girls | 100 | 5.25 | 14.31 | 1.43 |
| TOT d2 (TR - (O+C)) | boys | 66 | 337.47 | 68.72 | 8.45 |
|  | girls | 84 | 312.05 | 72.25 | 7.88 |
| CON d2 (TA - C) | boys | 66 | 133.68 | 39.43 | 4.85 |
|  | girls | 84 | 122.04 | 40.12 | 4.37 |

**Abbreviations:** TR. total number of responses; TA. total number of correct answers; O. omissions; C. commissions; TOT. total test effectiveness; CON. concentration index.

*Verbal fluency test.

**Supplemental Table 4.**

*Mean* *Comparison of attention, phonological and semantic fluency and speed of processing tests between sexes.*

| t-test for Equality of Means | | | | | | | |
| --- | --- | --- | --- | --- | --- | --- | --- |
|  |  |  |  |  |  | 95% CI of Difference | |
|  | t | df | Sig. (2 tailed) | Mean Difference | Std.Err.Dif | Lower | Upper |
| F* | -3.88 | 175 | 0.000 | -1.86 | 0.47 | -2.80 | -.091 |
| A* | -2.03 | 175 | 0.043 | -.94 | .46 | -1.86 | -0.03 |
| S * | -2.48 | 174 | 0.014 | -1.19 | .47 | -2.13 | -0.24 |
| Animals * | -1.56 | 175 | 0.120 | -.85 | .54 | -1.92 | 0.22 |
| Fruits* | -1.37 | 175 | 0.171 | -.53 | .39 | -1.30 | 0.23 |
| Speed Processing | -2.22 | 175 | 0.027 | -3.99 | 1.79 | -7.53 | -0.45 |
| TR (d2) | 2.31 | 175 | 0.022 | 27.94 | 12.07 | 4.10 | 51.77 |
| TA (d2) | 2.48 | 175 | 0.014 | 13.63 | 5.49 | 2.79 | 24.46 |
| O (d2) | -.06 | 175 | 0.952 | -.25 | 4.13 | -8.40 | 7.90 |
| C (d2) | .62 | 175 | 0.532 | 1.64 | 2.62 | -3.53 | 6.82 |
| TOT (d2) (TR - (O+C)) | 2.18 | 148 | 0.030 | 25.42 | 11.63 | 2.43 | 48.41 |
| CON (d2) (TA - C) | 1.77 | 148 | 0.077 | 11.64 | 6.55 | -1.29 | 24.59 |

**Abbreviations:** TR. total number of responses; TA. total number of correct answers; O. omissions; C. commissions; TOT. total test effectiveness; CON. concentration index.*Verbal fluency test.

**Supplemental Table 5**.

*Descriptives and multiple comparisons of grades in Spanish among different modalities of text presentation.*

| *Descriptives* | | | | | | | |
| --- | --- | --- | --- | --- | --- | --- | --- |
| Grades in Spanish | | | | | | | |
|  | N | Mean | Std. Deviation | Std. Error | 95% Confidence Interval for Mean | |  |
|  |  |  |  |  | Lower Bound | Upper Bound |  |
| visual | 64 | 6.19 | 1.75 | 0.219 | 5.75 | 6.63 |  |
| auditory | 53 | 6.51 | 1.68 | 0.231 | 6.05 | 6.97 |  |
| dual | 59 | 5.51 | 1.66 | 0.217 | 5.07 | 5.94 |  |
| Total | 176 | 6.06 | 1.74 | 0.131 | 5.80 | 6.32 |  |

| *ANOVA* | | | | | |
| --- | --- | --- | --- | --- | --- |
| Grades in Spanish | | | | | |
|  | Sum of Squares | df | Mean Square | F | Sig. |
| Between Groups | 29.69 | 2 | 14.84 | 5.119 | 0.007 |
| Within Groups | 501.74 | 173 | 2.90 |  |  |
| Total | 531.43 | 175 |  |  |  |

| *Multiple Comparisons* | | | | | | |
| --- | --- | --- | --- | --- | --- | --- |
| Dependent Variable: Grades in Spanish | | | | | | |
| Bonferroni | | | | | | |
| (I) Modality | (J) Modality | Mean Diff. | Std. Error | Sig. | 95% Confidence Interval | |
|  |  |  |  |  | Lower Bound | Upper Bound |
| visual | auditory | -0.32 | 0.316 | 0.931 | -1.09 | 0.44 |
|  | dual | 0.67 | 0.307 | 0.085 | -0.06 | 1.42 |
| auditory | visual | 0.32 | 0.316 | 0.931 | -0.44 | 1.09 |
|  | dual | 1.00^*^ | 0.322 | 0.007 | 0.22 | 1.78 |
| dual | visual | -0.67 | 0.307 | 0.085 | -1.42 | 0.06 |
|  | auditory | -1.00^*^ | 0.322 | 0.007 | -1.78 | -0.22 |
| *. The mean difference is significant at the 0.05 level. | | | | | | |

**SUPPLEMENTAL FIGURES**

**Supplemental Figure 1.**

*Correlation between grades in Spanish and verbal comprehension in boys and girls for different presentation modalities.*


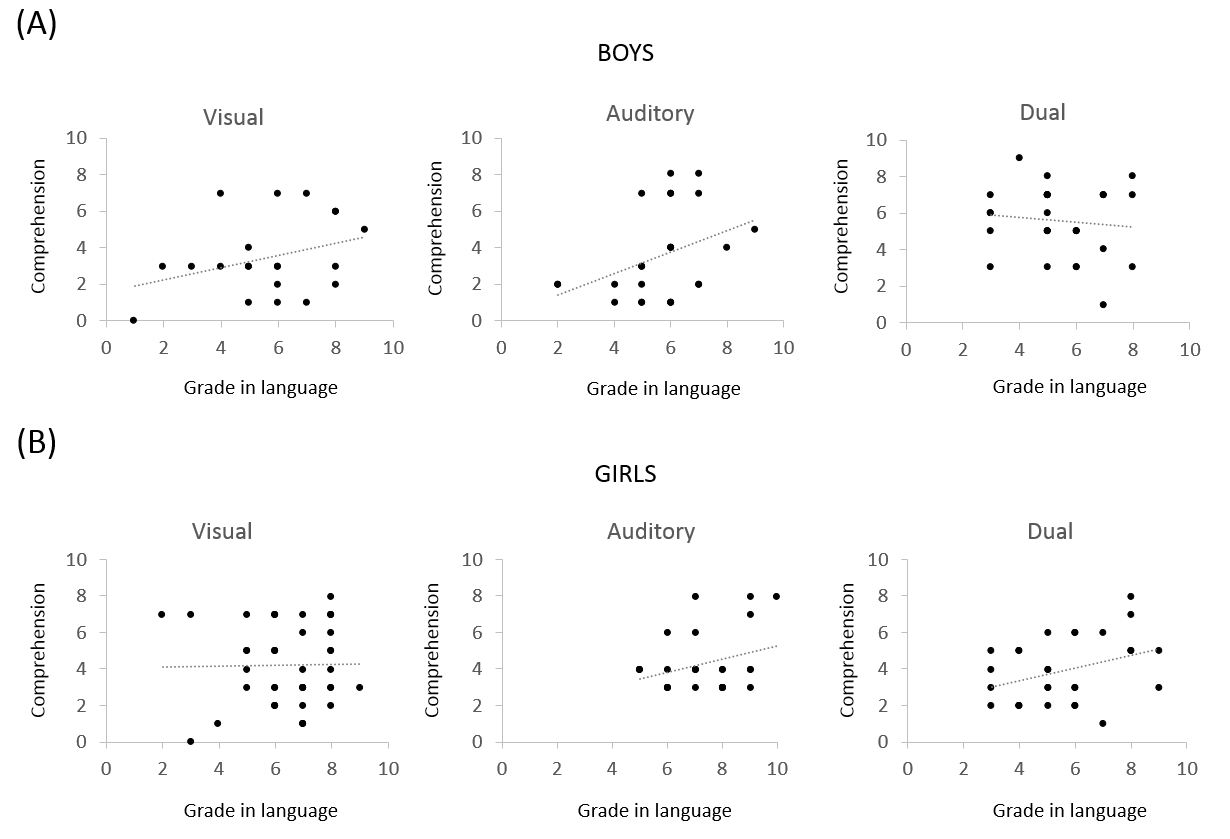


***Notes*:** A. Graphs showing individual data for text comprehension and grades in Spanish native language in boys. Every dot corresponds to a student. B. Same graphs for girls. Some dots overlap. Dotted lines are regression lines fitted to the experimental data.
